# The Periglomerular Cell of the Olfactory Bulb and its Role in Controlling Mitral Cell Spiking: A Computational Model

**DOI:** 10.1371/journal.pone.0056148

**Published:** 2013-02-06

**Authors:** Denise Arruda, Rodrigo Publio, Antonio C. Roque

**Affiliations:** Departamento de Física, Faculdade de Filosofia, Ciências e Letras de Ribeirão Preto, Universidade de São Paulo, Ribeirão Preto, Brazil; Georgia State University, United States of America

## Abstract

Interneurons in the olfactory bulb are key elements of odor processing but their roles have not yet being fully understood. Two types of inhibitory interneurons, periglomerular and granule cells, act at two different levels within the olfactory bulb and may have different roles in coordinating the spiking of mitral cells, which are the principal output neurons of the olfactory bulb. In this work we introduce a reduced compartmental model of the periglomerular cell and use it to investigate its role on mitral cell spiking in a model of an elementary cell triad composed of these two cell types plus a granule cell. Our simulation results show that the periglomerular cell is more effective in inhibiting the mitral cell than the granule cell. Based on our results we predict that periglomerular and granule cells have different roles in the control of mitral cell spiking. The periglomerular cell would be the only one capable of completely inhibiting the mitral cell, and the activity decrease of the mitral cell through this inhibitory action would occur in a stepwise fashion depending on parameters of the periglomerular and granule cells as well as on the relative times of arrival of external stimuli to the three cells. The major role of the granule cell would be to facilitate the inhibitory action of the periglomerular cell by enlarging the range of parameters of the periglomerular cell which correspond to complete inhibition of the mitral cell. The combined action of the two interneurons would thus provide an efficient way of controling the instantaneous value of the firing rate of the mitral cell.

## Introduction

The olfactory bulb is the first relay structure in olfactory processing. It receives direct input from olfactory receptor neurons in the olfactory epithelium and sends output to the olfactory cortex and other brain areas [1]–[3]. It also receives modulatory feedback input from higher brain areas [4].

The olfactory bulb has a complex internal circuitry [5]. There are two types of principal (output) excitatory neurons, mitral and tufted (M/T) cells, and two main inhibitory interneuron types, periglomerular (PG) and granule cells. The cell bodies and dendrites of these neurons are organized into layers. The most superficial layer is composed of structures called glomeruli, which are spherical tangles of receptor neuron axon terminals, dendrites of M/T cells and dendrites of PG cells. The somata of the latter neurons are located just outside glomeruli, hence their names. Within a glomerulus, the axons of receptor neurons make glutamatergic synapses with primary dendrites of M/T cells and PG cells [6]. The dendrites of PG cells form reciprocal dendrodendritic synapses with dendrites of M/T cells [6], [7]. Also, there is evidence that PG cells have self-inhibitory synapses (autapses) [8]. Each M/T cell has a single primary dendrite that extends apically towards the olfactory bulb surface and several secondary dendrites that spread laterally in the olfactory bulb [5]. Deeper within the olfactory bulb, at the so-called external plexiform layer, secondary dendrites of M/T cells make reciprocal dendrodentritic synapses with dendrites of granule cells.

Hence, there are two levels within the olfactory bulb at which inhibitory interactions occur. The roles of these two inhibitory circuits are not yet completely understood. In particular, it is not known how PG and granule cells coordinate their inhibitory interactions with M/T cells and how these affect the response properties of these cells [9]–[11].

A possible strategy to approach this problem is to put forth hypotheses to explain the role of each circuit element and to use data from experiments or theoretical models to verify them. Another strategy is to build detailed, data-constrained models of the cells and synapses involved and simulate circuits made of them. This can be done in a constructive way, starting with elementary microcircuits which can be grown to (scaled-down) versions of the whole network. Here we take the second strategy and construct a detailed simulation model of an elementary cell triad of the olfactory bulb made of a mitral, a periglomerular and a granule cell.

To construct our model, we need detailed models of the three cells involved. There are many compartmental conductance-based models of mitral and granule cells availabe [12]–[20] but, to our knowledge, there is no model of such a kind of the PG cell.

In this work we present a multicompartmental conductance-based model of the PG cell fitted according to available experimental data [5], [8], [21]–[27] and inspired on a model of the glomerulus circuitry [28]. This model was combined with already existing conductance-based models of mitral [14] and granule cells [15] available at ModelDB [29] to construct our elementary cell triad model. This model was used to investigate the role of the two inhibitory interneurons on the firing rate of the mitral cell.

The main parameters investigated in our simulations were the conductances of the dendrodendritic synapses, the amplitudes, durations and onset times of excitatory current pulses applied to the mitral and PG cells representing stimuli coming from olfactory receptor neurons, and the amplitude, duration and onset time of an excitatory current pulse applied to the granule cell representing the average input from other cells of the olfactory bulb. We performed simulations in which these parameters were varied and studied their effects on the spiking behavior of the mitral cell.

Our simulation results allow us to predict different roles for the PG and granule cells in controlling the firing rate of mitral cells. We hope our model may be helpful to understand the coordinated effect of these interneurons on the temporal processing of information within the olfactory bulb.

## Results

### Periglomerular Cell Model

**Figure 1:**
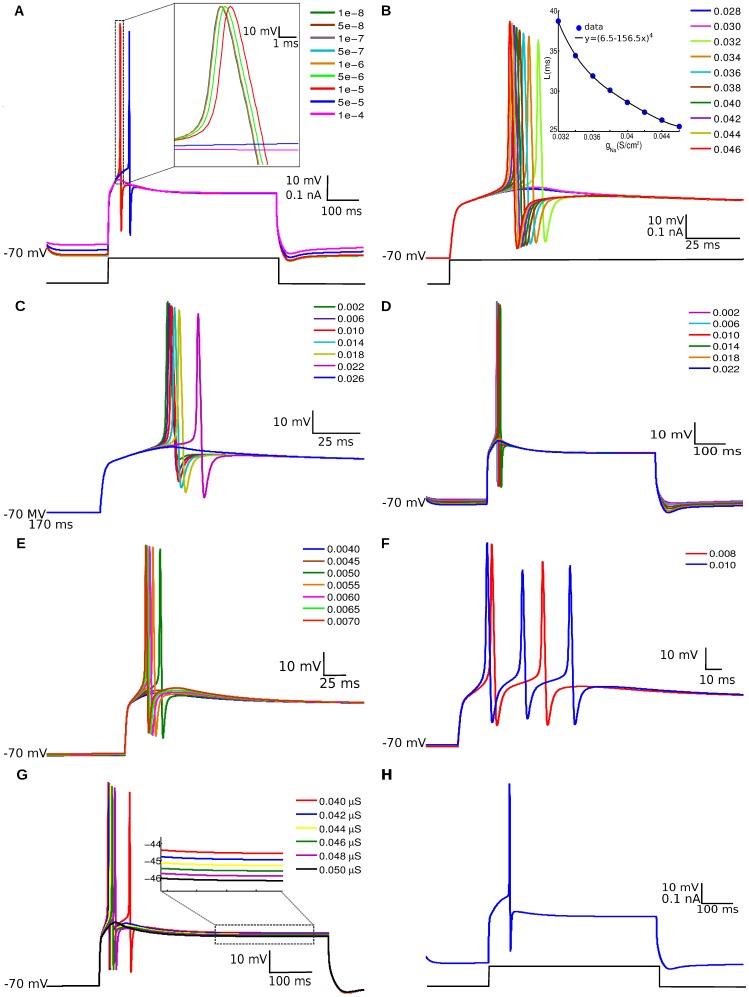
Effect of ionic conductances on the response of the PG cell model. The graphs show the membrane potential of the PG cell model in response to a depolarizing current of 100 pA injected at the soma for 600 ms starting at 200 ms. The maximum conductance densities (in S/cm^2^) are indicated in each graph. (A) Effect of varying the maximum conductance density of the hiperpolarization-activated current (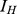). A zoom of the region indicated by dots is shown at the inset. (B) Effect of varying the maximum conductance density of the sodium current (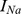). The inset shows the latency (L) of the spike peak measured from stimulus onset as a function of 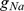. (C) Effect of varying the maximum conductance density of the rectifier potassium current (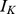). (D) Effect of varying the maximum conductance density of the A-type inactivating potassium current (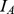). (E) Effect of varying the maximum conductance density of the T-type calcium current (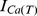). (F) Effect of values of the 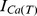 current higher than 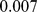 S/cm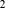. The graph shows the behavior of the membrane potential for two values of 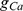 (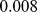 S/cm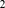 and 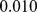 S/cm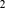). (G) Effect of varying the maximum conductance of the self-inhibitory synapse on the membrane potential of the PG cell model. (H) Response of the PG cell model with the parameters of table 1.

The maximum conductance densities of the ionic currents in the PG cell model (described in Materials and Methods) were varied in order to verify the effect of each ionic current on the PG cell response. Figure 1 shows the effect of varying each one of the maximum conductance densities of the PG cell on its firing behavior while the other conductances are kept at fixed values. In every case the range of parameter variation was centered at the value given in the original ionic current model (default value) described in Materials and Methods. The stimulation protocol used to generate the graphs was the same for all cases, namely a depolarizing current of 100 pA was injected at the soma for 600 ms.

Figure 1A shows the effect of varying the maximum conductance density of the hyperpolarization-activated current (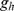) between 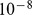 and 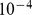 S/cm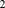. Notice that when 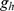 is above 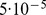 S/cm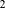 the resting potential is shifted upwards from the default value (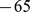 mV). For 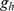 smaller than 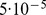 S/cm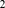 the injected current produces a single spike and the lower the value of 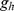 the smaller the delay between stimulus onset and spike peak. For 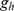 above 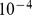 S/cm^2^ there is no spike.

The effects of variations in the maximum conductance densities of the Hodgkin-Huxley-like sodium (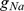) and potassium (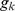) currents are presented in figures 1B and 1C, respectively. In figure 1B one can see the strong influence of the sodium current in shaping the action potential. For 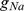 below 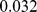 S/cm there is no spike. From 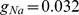 S/cm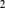 onwards there is a spike and the higher the value of 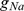 the earlier it occurs. The inset shows the latency of the spike peak measured from stimulus onset as a function of 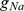. The best fit curve is a fourth order polynomial. The potassium current, on the other hand, has the opposite effect. Figure 1C shows that the smaller the value 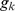 the earlier the spike occurrence. In particular, when 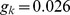 S/cm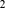 the cell does not fire. Notice also that the after-spike hyperpolarizing rebound gets deeper as 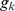 increases.

The effect of the A-type inactivating potassium current can be seen in figure 1D, where it is possible to observe that the lower its maximum conductance density (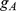), the smaller the firing delay. For 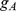 above 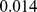 S/cm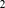 there is no spike.

Figures 1E and 1F show the effect of the maximum conductance density of the T-type calcium current. This transient current is responsible for the low threshold spike (LTS) of the PG cell [24]. As 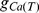 increases, the faster the cell fires and the higher the afterhyperpolarization rebound. For 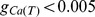 S/cm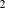 the cell model does not spike. On the other hand, for 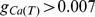 S/cm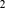 (see figure 1F), the cell fires more than one spike. This behavior is observed experimentally [24].

Figure 1G shows the effect of varying the maximum conductance (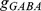) of the self-inhibitory synapse incorporated into the PG cell model. For 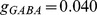 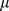
S the cell fires two spikes and for values of 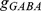 above this value the cell fires a single spike with increasing delay. For 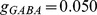 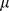
S the model does not fire. Besides affecting spiking, 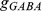 also affects the afterhyperpolarization plateau (see inset), with higher values of 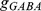 reducing the plateau level.

After determining the effect of each ionic current on the electrophysiological response of the PG cell, we made a parameter search (as described in Materials and Methods) to adjust our PG cell model in comparison with experimentally available data [24]. The stimulation protocol, also taken from [24], was the same used in the analyses described above. The fit took into consideration spike amplitude, spike time and the potential value at the afterhyperpolarization plateau. The search considered conjoint variations of all the conductance densities mentioned above plus the synaptic conductance of the self-inhibitory autapse. The variations were restricted to the ranges presented above. The most suitable combination of parameters is given in Table 1, and the corresponding response of our PG cell model is given in Figure 1H. This response reproduces qualitatively the experimentally found behavior of the cell (figure 1C of [24]), which corresponds to the firing of a single action potential followed by a membrane potential plateau. A comparison between the values of spike peak, spike time and plateau level for our model and the real cell is given in Table 2.

**Table 1:** Best parameter values for the PG cell model.

	Maximum Conductances (nS)				
Compartment	*G_H_*	*G_K_*	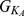	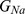	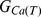
soma	0.101	44.420	30.950	61.850	9.723
axon	0.079	34.690	24.180	48.310	7.595
dendrites	0.031	13.880	9.671	19.320	3.038
dendritic shaft	0.002	0.694	0.0484	0.966	0.152
gemmule	0.002	0.694	0.0484	0.966	0.152
	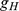	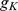	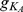	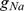	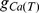
**Maximum conductance** **densities (S/cm 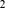 )**	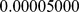	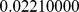	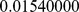	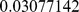	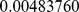
**Maximum synaptic** **conductance ( 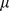 S)**			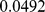		

The conductance densities and the synaptic conductance were obtained from equation (1) with an error of 0.01094.

**Table 2:** Comparison between parameters of the PG cell model and the real one.

Parameter	Model	Real cell
spike peak	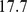 mV	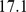 mV
spike time	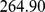 ms	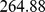 ms
plateau	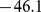 mV	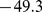 mV

Parameters of the real cell taken from McQuiston et al. [24].

### Mitral and Granule Cell Models

The mitral cell model described in Materials and Methods was implemented by us and tested for consistency in comparison with data from the original implementation of the model [30]. The model was subjected to the same stimulation protocol described in [30], in which a current injection of 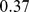 nA is applied for 400 ms with onset at 50 ms in the glomerular tuft compartment. This protocol is compatible with experiments of current injection in mitral cells [31].

**Figure 2:**
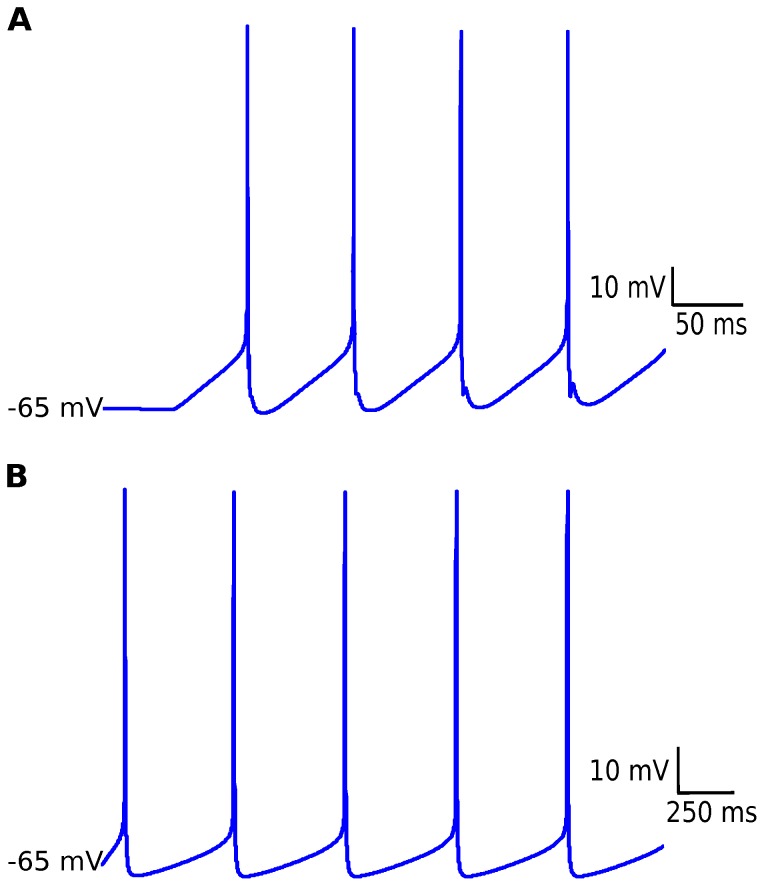
Responses of the mitral and granule cell models. (A) Membrane potential of the mitral cell model in response to a depolarizing current injection of 0.37 nA for 400 ms. (B) Membrane potential of the granule cell model in response to a depolarizing current injection of 0.0625 nA for 2500 ms. In both curves the stimulation protocol used by Davison [30] was reproduced.

The response of our implementation of the mitral cell model is given in Figure 2A, and can be compared with the similar response shown in [30]. They are identical.

The same procedure was done for the granule cell model, wich was stimulated with a depolarizing current injection of 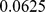 nA for 2500 ms starting at 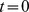 [30]. The response of our granule cell model implementation is given in (Figure 2B) and it is also identical to the one shown in the original model [30].

### Coupled Mitral and PG Cells

**Figure 3:**
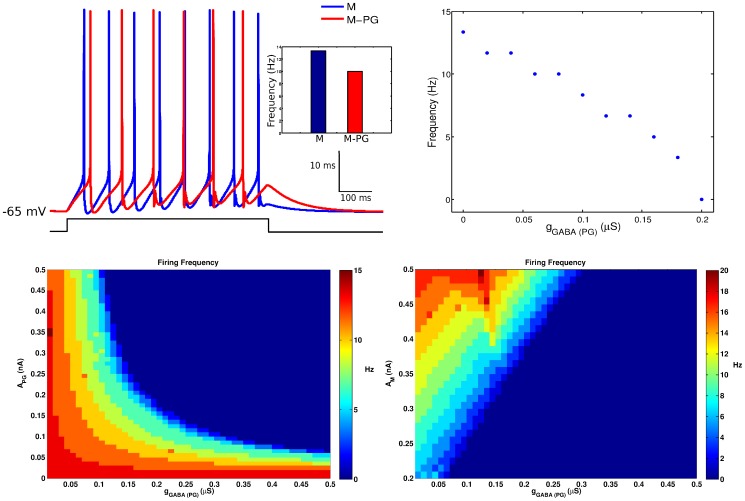
Effect of inhibition from the PG cell on the response of the mitral cell. (A) Membrane potential of the mitral cell in response to hyperpolarizing current pulses of 0.18 nA and 0.37 nA injected into the PG and mitral cells respectively, for 600 ms with the same onset time of 50 ms. The histogram shows the firing frequency of the mitral cell in the absence (blue) and presence (red) of the inhibitory synapse. (B) Mitral cell’s firing frequency as a function of the maximum synaptic conductance 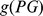 of the inhibitory synapse made by the PG cell. (C) Diagram showing the firing frequency of the mitral cell, indicated in color code according to scale in sidebar, as a function of the maximum synaptic conductance 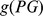 and the amplitude of the current pulse injected into de PG cell (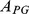). (D) Diagram showing the firing frequency of the mitral cell, indicated in color code according to scale in sidebar, as a function of the maximum synaptic conductance 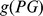 and the amplitude of the current pulse injected into de mitral cell (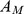).

As an initial investigation, we considered only the mitral and the PG cells and studied the effect of the reciprocal dendrodendritic coupling between them (see Materials and Methods) on the response of the mitral cell. Initially, the isolated mitral cell was submitted to a current pulse of 0.37 nA for 600 ms beginning in 50 ms. Then, after coupling the two cells, we submitted the mitral cell to the same current pulse and the PG cell to a current pulse of same duration and onset but amplitude of 0.18 nA. These current pulses represent inputs from receptor neurons. The maximum conductances of the excitatory synapse from the mitral cell onto the PG cell and of the inhibitory synapse from the PG cell onto the mitral cell were the same (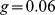 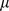
S). The responses of the mitral cell in isolation and after coupling are shown in Figure 3A. The presence of the PG cell increases the latency of the first spike and the interspike interval of the mitral cell. Therefore, it reduces the firing frequency of the mitral cell. The effect of the maximum conductance of the PG cell inhibitory synapse (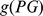) on the firing frequency of the mitral cell is given in Figure 3B. The frequency decays in an approximately linear fashion as a function of 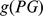.

We also analyzed the combined effect of 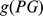 and the amplitudes of the stimuli injected into the PG (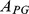) and mitral (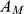) cells. The diagram of Figure 3C shows the firing frequency of the mitral cell as a function of 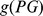 and 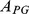 for a fixed value of 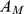 (the same as above). The diagram shows approximately symmetric roles for the two parameters. There is a range of values of 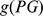 sufficiently small that the mitral cell cannot be completely silenced no matter how strongly the PG cell is stimulated. Similarly, there is a range of sufficiently small stimuli to the PG cell 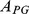 for which even very high inhibitory conductances 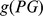 cannot completely silence the mitral cell. On the other hand, there is a large region in the parameter space (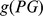-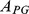), corresponding to high values of both parameters, for which the firing frequency of the mitral cell is zero. The diagram in Figure 3D shows the firing frequency of the mitral cell as a function of 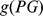 and 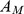 for a fixed value of 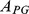 (0.18 nA as in Figures 3A and B). There is no symmetry in this case. The PG cell inhibition has a stronger effect on the mitral cell spiking than the amplitude of the excitatory stimuli received by the mitral cell from sensory neurons. For low 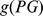 values, increasingly higher 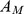 values can put the mitral cell in higher spike frequency regimes, but for a relatively large range of 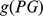 values the mitral cell does not spike even for strong excitatory inputs.

**Figure 4:**
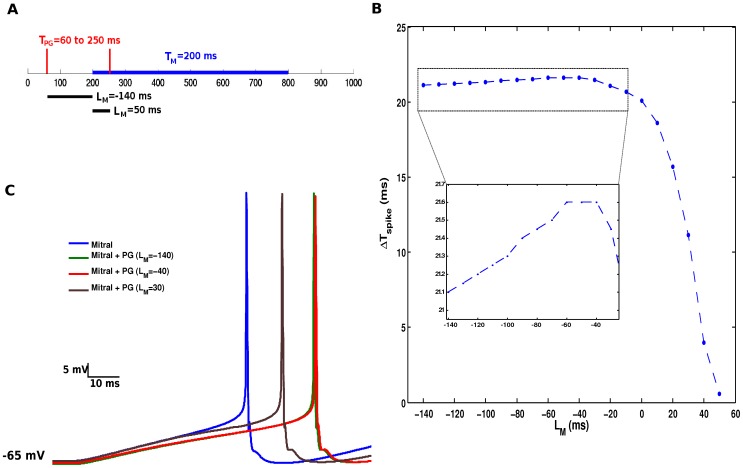
Time interval between arrival of stimuli to PG and mitral cells and its effect on the first spike of the mitral cell. (A) Scheme showing the times of arrival and durations of the external stimuli applied to PG and mitral cells. The blue bar indicates the mitral cell’s stimulus duration with onset at 200 ms. The red vertical bars indicate the range of values of the PG cell’s stimulus onset time and the black bars indicate the corresponding range of values for the interval 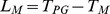. (B) Difference between the time of the first spike of the mitral cell when coupled to the PG cell and in isolation, denoted by 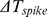, as a function of interval 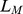. A zoom on the rectangular area surrounded by dots is shown in the inset. (C) Time course of the membrane potential of the mitral cell when in isolation (leftmost curve) and for three different values of the interval 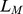.

In our simulations, the difference between the onset times of injected current pulses into the mitral cell (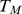) and the PG cell (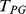 was defined as the interval 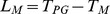 (see Figure 4A). We investigated the effect of 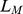 on the time of the first spike of the mitral cell in comparison with the time of its first spike when isolated. Initially, we measured the time of first spike of the isolated mitral cell when it was submitted to an injected current pulse of 0.37 nA for 600 ms starting at 200 ms. This time was 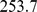 ms. Then, we coupled the mitral cell to the PG cell and the former received the same external stimulus with the same onset and duration as in isolation while the latter received an injected current pulse of 0.18 nA with onset times ranging from 60 ms to 250 ms. The duration of the stimulus applied to the PG cell varied from one experiment to the other so that it always ended together with the stimulus applied to the mitral cell. In this investigation, the maximum conductances of the dendrodendritic synapses were kept at 0.06 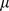
S. Figure 4B shows the difference between the time of first spike of the mitral cell (measured from 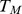) when it is coupled to the PG cell and when it is uncoupled, 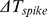, as a function of the interval 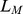. The inset shows a detail of this curve for −140 ms 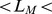 −20 ms. The value of 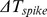 grows steadily towards a maximum plateau for −60 ms 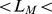 −40 ms and then decays rapidly towards zero for 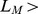 −40 ms. This shows that there is an optimum interval between the arrival of stimuli to the PG cell and the mitral cell for which the increase in the latency of the first spike of the mitral cell provoked by inhibition from the PG cell is maximal. The effect of the PG cell on the latency of the first spike of the mitral cell is slighly suboptimal when the two cells are stimulated simultaneously (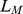 = 0). Notice that even for a range of positive 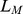 values, in which the mitral cell receives its external stimulus before the PG cell, the PG cell spike causes an increase in the latency of the first spike of the mitral cell in comparison with the isolated mitral cell case. Figure 4C shows the mitral cell responses when it is isolated and for three different values of 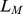. From right to left, the first trace corresponds to the largest spike latency, the second one (very close to the first) corresponds to a suboptimal case, the third corresponds to a case in which the external stimulus arrives first on the mitral cell than on the PG cell, and the fourth trace is for the uncoupled mitral cell.

### Cell Triad

**Figure 5:**
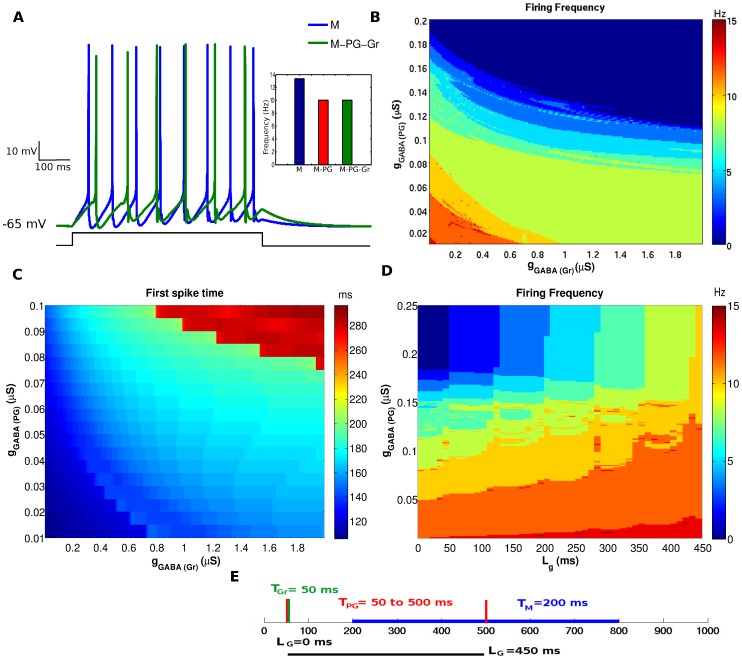
Combined effect of the inhibitory synapses from the PG and granule cells on the mitral cell’s response. (A) Membrane potential of the mitral cell in response to hyperpolarizing current pulses of 0.18 nA, 0.37 nA and 0.0625 nA injected into the PG, mitral and granule cells respectively, for 600 ms with the same onset time of 50 ms. The inset shows the histogram of the mitral cell’s firing frequency when it is isolated (M, indicated in blue), when it is coupled only to the PG cell (M-PG, indicated in red) and when it is coupled to the PG and granule cells (M-PG-Gr, indicated in green). (B) Diagram showing the firing frequency of the mitral cell, indicated in color code according to scale in sidebar, as a function of the maximum synaptic conductances of the inhibitory synapses made into it by the granule (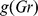) and PG (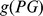) cells. (C) Diagram showing the time of first spike of the mitral cell, indicated in color code according to scale in sidebar, as a function of the maximum synaptic conductances 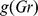 and PG 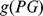. (D) Diagram showing the firing frequency of the mitral cell, indicated in color code according to sidebar, as a function of the interval 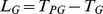 between onsets of external stimuli to the PG and granule cells and the maximum synaptic conductance 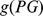. (E) Scheme showing the times of arrival and durations of the external stimuli applied to the PG, the granule and the mitral cells. The blue bar indicates the mitral cell’s stimulus duration with onset at 200 ms. The red vertical bars indicate the range of values of the PG cell’s stimulus onset time and the green vertical bar indicates the stimulus onset time of the granue cell. The black bar underneath indicates the range of values of the interval 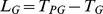.

The model of the cell triad was constructed by adding the granule cell model to the coupled mitral and PG cell models presented in the previous section. This was done by coupling the granule and mitral cell models via reciprocal dendrodendritic synapses as described in Materials and Methods. In an initial study, we set the maximum conductance as 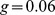 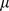
S for all synapses in this small circuit. The current pulses injected into the PG and mitral cells were the same as used in the previous section and the granule cell received a depolarizing current injection of 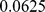 nA, representing average inputs from other OB cells. The current pulses were applied to all three cells with the same duration of 600 ms and same onset time of 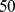 ms. Figure 5A shows the responses of the mitral cell in isolation and when coupled to the PG and granule cells. The inset in this figure compares the firing frequency of the mitral cell when it is isolated, when it is coupled only to the PG cell and when it is coupled to both the PG and granule cells. The firing frequency of the mitral cell is reduced when it is inserted into the cell triad but the reduction is the same as for the case in which it is coupled only to the PG cell. This shows that, for the conditions of this study, the effect of adding the granule cell to the coupled mitral and PG cells on the firing frequency of the mitral cell is negligible. This suggests that the firing frequency of the mitral cell is controlled upstream by the PG cell.

The first study described above is too restrictive because it imposes the same value for all the maximum conductances in the cell triad model. To investigate the effect of different combinations of 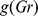 and 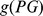 on the firing frequency of the mitral cell we conducted another study in which the values of these two parameters were sistematically varied. The results are shown in the diagram of Figure 5B. It shows that the roles of the two inhibitory cells are not symmetric. Only the PG cell has absolute power to make the mitral cell stop firing: for high values of 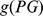 (close to 0.2 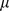
S), the firing frequency of the mitral cell is zero independently of the 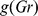 value. On the other hand, for high values of 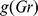 (above 1 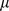
S), the firing frequency of the mitral cell is organized into more or less horizontal bands in the 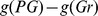 diagram and the smallest frequency of these bands corresponds to about half of the maximum frequency of the mitral cell. Since the bands are horizontal, in this large region of the 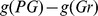 diagram the firing frequency is much more sensitive to changes in 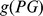 than in 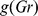. Depending of the value of 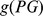 the frequency of the mitral cell is confined to one of these bands and changes in 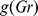 can little affect this frequency. The strongest relative effect of the granule cell occurs for low values of 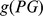 (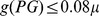
S). In this part of the 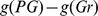 diagram the roles of 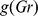 and 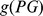 become more symmetric and an increase of 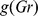 can reduce the firing frequency of the mitral cell to about half of its maximum value. This relative increase of the inhibitory role of the granule cell is due to the weak inhibition of the mitral cell by the PG cell. Since the mitral cell is more active in this case it evokes a stronger recurrent inhibition from the granule cell, which in turn reduces the mitral cell’s activity. However, the inhibition done by the granule cell is never as efficient as the one done by the PG cell.

Figure 5C is a diagram giving the time of first spike of the mitral cell in terms of 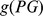 and 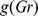. In the absence of inhibition, the mitral cell fires the first spike around 103 ms. When the inhibitory synapses are added and their maximum conductances are increased, the latency to the first spike becomes increasingly larger. The roles of the PG cell and granule cell in controlling the latency to the first spike of the mitral cell are more symmetric than in the case of controlling the firing frequency of the mitral cell but the inhibition from the PG cell has a slighly larger influence on the latency to first spike time than the inhibition from the granule cell, especially for longer latencies. For high values of 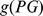 (close to 0.1 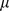
S) the latency is at its peak irrespective of the value of 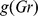 for more than half of the range of possible 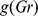 values. On the other hand, for mid to high values of 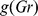 (above 0.8 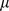
S) a considerable range (more than half) of 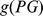 values can prevent the latency to reach its peak.

Another study done by us was on the influence of the time interval between the onset of current injection in the PG cell (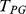) and the onset of current injection into the granule cell (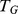), represented by 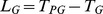, on the firing frequency of the mitral cell. In this study, the mitral cell was submitted to an injected current pulse starting at 200 ms and with duration of 600 ms; the granule cell was submitted to an injected current pulse starting at 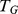 = 50 ms and lasting until the end of the pulse applied to the mitral cell (800 ms); and the PG cell was submitted to an injected current pulse with variable onset time (in the range 50 ms 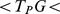 500 ms) which also lasted until 800 ms. In this study, the maximum conductance of the inhibitory synapse from the granule cell onto the mitral cell was maintained at 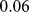 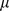
S and the maximum conductance of the inhibitory synapse from the PG cell onto the mitral cell was varied conjointly with the interval 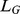. The reason for this was to evaluate the combined effect of intrinsic and extrinsic parameters of the PG cell on the response of the mitral cell. The diagram of the firing frquency of the mitral cell as a function of 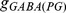 and 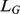 is given in Figure 5D and a scheme of the onset times and durations of stimuli applied to the mitral, granule and PG cells is given in Figure 5E.

For simultaneous arrival of stimuli to the PG and granule cells (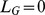) the diagram of Figure 5D shows the already known result that the firing frequency of the mitral cell is reduced in a stepwise fashion by increasing the maximum conductance of the inhibitory synapse made by the PG cell, 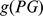: the leftmost vertical line of the diagram is divided into bands of 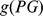 values for which the mitral cell’s firing frequency are constant, and as 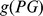 grows along this line the firing frequency of the mitral cell decreases through jumps from one band to another. Notice that when 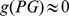 along this line the firing frequency of the mitral cell is close to maximal, indicating that the granule cell is not effective to inhibit the mitral cell (for the maximum conductance of its inhibitory synapse set to 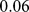 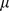
S). This result is consistent with the result shown in Figure 5A. For 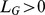, as one moves to the right in the 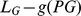 diagram the inhibitory influence of the PG cell over the mitral cell gets weaker as reflected by the increase in the widths of the high frequency bands (see the lower part of the diagram). In the upper part of the diagram, when 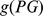 is high, the low frequency bands even bend upwards so that as 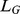 increases, and the inhibitory influence of the PG cell decreases, the firing frequency of the mitral cell grows in a stepwise fashion as in the case for 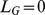.

**Figure 6:**
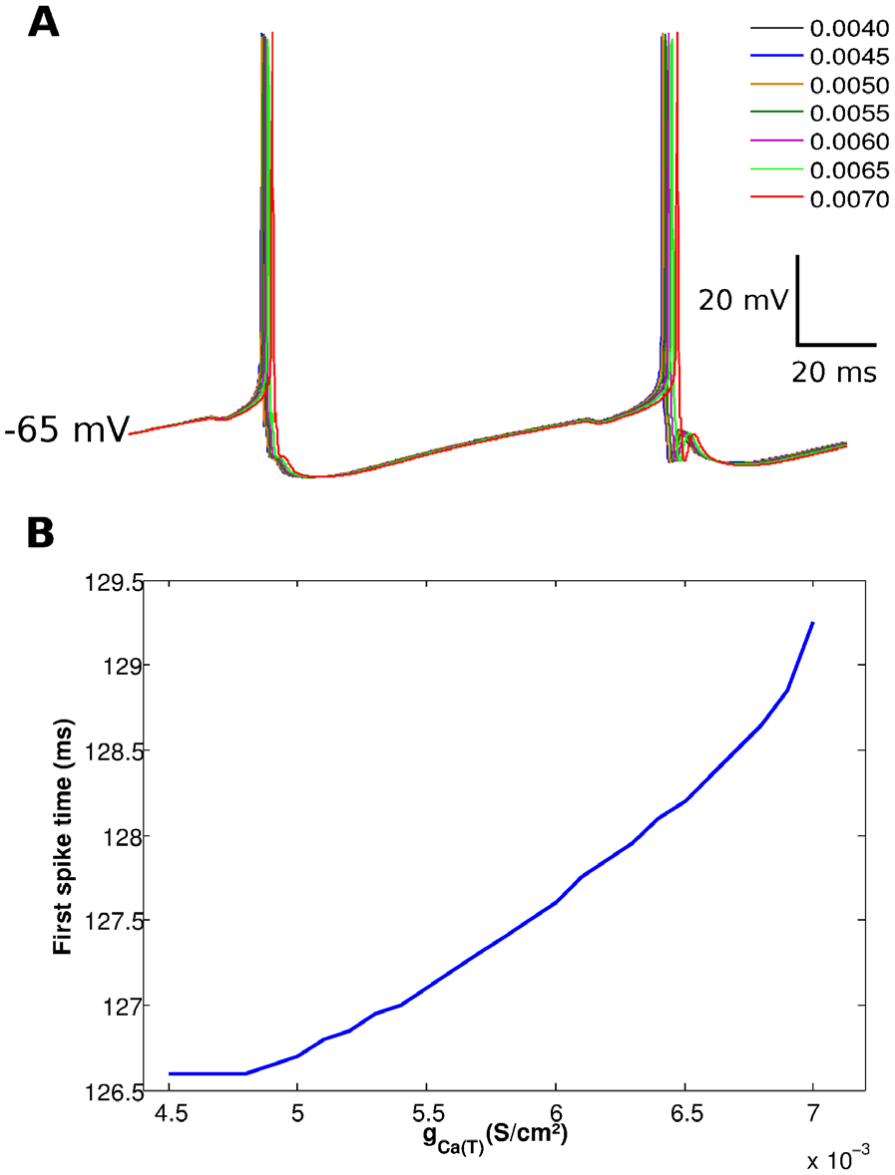
Effect of the maximum conductance density of the calcium current of the PG cell on the response of the mitral cell. (A) Curve of the membrane potential of the mitral cell *versus* time for different values of the maximum conductance density of the T-type calcium current, 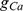. The color-coded values are indicated in the upper right corner of the figure. (B) Time of first spike of the mitral cell as a function of 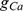.

We also used our cell triad model to investigated how active membrane parameters of the PG cell could influence the mitral cell response. Out of all the PG cell active parameters, the most significant changes on mitral cell spiking were provoked by the maximum conductance density of the T-type calcium current, 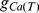. Figure 6A shows the effect of changes in 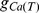 on the membrane potential of the mitral cell. The increase of 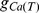 delays the action potentials of the mitral cell slightly. The effect of 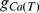 on the time of first spike of the mitral cell is shown in Figure 6B. It shows that the latency to first spike increases with the increase of 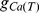.

## Discussion

In this work we introduced a multicompartmental conductance-based model of the PG cell of the olfactory bulb. The construction of the model was based on experimental morphological and electrophysiological data [5], [8], [21]–[27] and on a previous modeling work [28]. The characteristics and properties taken into account to construct the PG cell model were sufficient to produce a response curve (see Figure 1H) corresponding to the observed electrophysiological behavior under the same experimental conditions [24].

We also studied the effect of each ionic current present in the model on the response curve by varying their maximum conductance densities. This allowed us to identify the conductance parameters that most influenced the response of the PG cell. They are the maximum conductance densities of the sodium and T-type calcium currents and the maximum conductance of the self-inhibitory synapse. In particular, the T-type calcium current influences the spike time and the spike shape and controls the afterhyperpolarization rebound. For very high values of the maximum conductance density 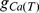 the cell fired more than one spike (Figure 1G). The explanation for this is that the calcium current is activated by low voltage values, so the afterhyperpolarization voltage resulting from the high 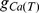 value was sufficient to generate one or more spikes. This effect shows that the T-type calcium current acts on the subthreshold activity of the PG cell and controls the ocurrence of repetitive spikes.

The objective of constructing the compartmental model of the PG cell was to use it together with availabe compartmental models of the mitral and granule cells to construct a model of the basic cell triad of the olfactory bulb. This model was used here in a series of *in silico* studies to investigate the relative roles of the PG and granule cells of the spiking behavior of the mitral cell. In the future, this elementary cell triad model can be used in the construction of large-scale models of the olfactory bulb.

In the first study performed by us the granule cell was disconnected from the cell triad so that we could study the inhibitory effect of the PG cell alone on the mitral cell. As expected, the action of the PG cell results in a reduction of the firing frequency and an increase of the latency to the first spike of the mitral cell. Our results (Figure 3) show that the PG cell can be very efficient in reducing the firing frequency of the mitral cell. This efficiency can be governed by two parameters: the maximum conductance of the inhibitory synapse made by the PG cell and the strength of the stimulus arriving at the PG cell, which in our case was represented by the amplitude of the current pulse injected into it. Even when the mitral cell is excited by a strong stimulus the PG cell is capable of completely inhibiting the mitral cell (for high values of the maximum conductance of its synapse) or simply reduce the firing frequency. This suggests that the shape of the stimulus-response curve of the mitral cell can be controlled by the inhibitory action of the PG cell alone. The control of the first spike time of the mitral cell by the PG cell depends on the times at which the two cells start being simulated by the injected current pulses. This suggests that the relative arrival times of odorant stimuli to the PG and mitral cells are important in the regulation of the response of the olfactory bulb to these stimuli.

Interestingly, when the granule cell was added to the model by coupling it via dendrodendritic synapses with the mitral cell our results showed that the granule cell had little effect on the firing frequency of the mitral cell. The main result of this work is the one contained in the diagram of Figure 5B. It shows that for most of the possible combinations of the maximum synaptic conductances of the PG and granule cells the major controller of the mitral cell’s firing frequency is the PG cell. For some values of its maximum synaptic conductance the PG cell is the sole controller of the mitral cell’s firing frequency, being capable to silence the mitral cell independently of the granule cell. This only happens to the granule cell when the maximum conductance of the PG cell synapse is zero, and the most that the granule cell can do in this case is to reduce the firing frequency of the mitral cell to about half of its maximum frequency. Therefore, the PG cell is the only one of the two inhibitory interneurons considered here that is effective in completely inhibiting the mitral cell.

An analysis of the diagram of Figure 5B suggests possible different roles for the periglomerular and granule cells in the control of mitral cell firing activity, in the case of the isolated cell triad considered here. In the absence of inhibition by the granule cell (leftmost vertical line in the diagram, corresponding to 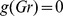), the firing frequency of the mitral cell is organized into frequency bands going from zero frequency to maximum frequency. As one moves to the right in the diagram and the inhibitory action of the granule cell gradually increases, the width of the zero frequency band grows so that the range of 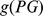 values which correspond to complete inhibition of the mitral cell grows as well. On the other hand, the widths of the high frequency bands are not reduced but these bands are displaced to the region of very low values of 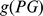 in the diagram and eventually disappear. These all suggest that the effect of the PG cell, apart from the capability of silencing the mitral cell, would be to provide a stepwise control of the mitral cell activity, and the effect of the granule cell would be to facilitate the inhibitory action of the PG cell by enlarging the step size, i.e. the range of 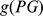 values which correspond to a given frequency of the mitral cell.

The relative time moments at which the cells in the cell triad start being stimulated by external stimuli are also important for the firing frequency of the mitral cell. For example, our results for a particular value of the maximum synaptic conductance of the granule cell synapse show that the inhibitory influence of the PG cell on the mitral cell gets weaker as the onset time of current injection in the PG cell gets closer to the onset time of current injection in the mitral cell than to the onset time of current injection in the granule cell (as measured by 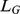 in Figure 5D). A possible explanation for this is that the summed up inhibitory effect of the PG cell over the mitral cell is reduced as 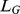 increases. For 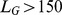 ms the PG cell even starts to be stimulated by the current pulse after the mitral cell. Interestingly, for large values of 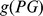 (upper part of the diagram in Figure 5D) the stepwise variation of the mitral cell’s firing rate is controlled by 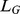. Based on the result shown in Figure 5B it is safe to assume that this result is valid for most values of 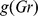 and not only for the one chosen to generate the diagram of Figure 5D). Hence, our results suggest that in the case of strong inhibitory synapse from the PG cell into the mitral cell it is the relative timing of arrival of external stimuli to the PG and mitral cells that determine the efficiency of the PG cell in inhibiting the mitral cell. An earlier arrival in the PG cell than in the mitral cell could result in complete inhibition while a later arrival in the PG cell than in the mitral cell would result only in partial inhibition.

Our results also show that intrinsic membrane mechanisms of the PG cell may influence the firing behavior of the mitral cell. In particular, we studied the effect of the maximum conductance density of the T-type calcium current and showed that an increase in this maximum conductance results in delaying the time of first spike of the mtral cell. This effect can be explained by the action of the T-type calcium current on the subthreshold activity of the PG cell as commented above. Large values of 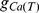 make it easier for the PG cell to spike and thus increase the efficiency of this cell in inhibiting the mitral cell.

The above discussion was based on what can be inferred from the results of our studies. They show that the PG cell can be highly effective in preventing or reducing the mitral cell spiking and illustrate how the interactions between the inhibitory synaptic inputs of the PG and granule cells on the mitral cell can regulate the firing frequency and the spike time of the mitral cell. They also show that the firing behavior of the mitral cell depends on the strengths and onset times of the inputs received by the cells in the triad. How can these results be interpreted in terms of current hypotheses on the roles of inhibitory interneurons in the olfactory bulb?

Inhibitory neurons in the olfactory bulb are thought to mediate lateral inhibitory circuits, which subserve contrast enhancement by reducing the degree of correlation between cells activated by simular stimuli [10], [11], [32]. Due to the high-dimensionality of olfactory feature space contrast enhancement in the olfactory bulb has to rely on mechanisms different than the neighborhood-based ones found in, e.g. the early visual or somatosensory systems [33]–[35]. Cleland and coworkers have recently proposed a mechanism of contrast enhancement in the olfactory bulb that does not depend on physical proximity between glomeruli [11], [28], [35]. This mechanism relies on the local inhibitory interactions between PG and mitral cells whithin a glomerulus and requires that PG cells be more sensitive to common afferent input from olfactory receptor neurons than their coglomerular mitral cells. The models by Cleland and coworkers show that the response curve of a mitral cell in a glomerulus as a function of input strength (representing odorant features) has a half-hat profile. Weak inputs that activate more strongly the PG cell than the mitral cell do not evoke a net response of the mitral cell while strong inputs do. When combined with responses from mitral cells associated to other glomeruli, these half-hats combine to form the characteristic Mexican hat profile observed experimentally [36].

Our results, albeit restricted to a single cell triad and ignoring network effects, are compatible with the model of Cleland and coworkers. The results of our study on the coupled mitral and PG cells alone (Figure 3) show that the inhibition of the PG cell controls the mitral cell’s sensitivity (the minimum input value from which the mitral cell starts firing) and maximum firing rate, i.e. the PG cell controls the response curve of the mitral cell. In particular, observing Figure 3D one can see that, for a range of moderate 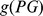 values, the firing rate of the mitral cell as a function of input amplitude has a half-hat-like profile. The PG cell capability to fully silence the mitral cell found in our model is also compatible with experimental evidence that PG cells can completely prevent glomerular activity [34]. As a novelty regarding the models of Cleland and coworkers, which did not consider the granule cell, our results show that the early control of the mitral cell spiking provided by the PG cell is largely unperturbed by the granule cell. Only when the strength of the inhibitory synapse of the PG cell is small the inhibitory action of the granule cell becomes significant, due to the granule cell excitation by the now more active mitral cell. This is a type of activity-dependent inhibition [10]. However, at least in the context of our cell triad model, the granule cell cannot silence the mitral cell completely to shape its response profile into a half-hat as done by the PG cell. It remains possible, though, that in a network context the granule cell is so strongly activated by other mitral cells that its lateral inhibitory action upon the mitral cell be enough to silence it.

Another interesting result of our model is the stepwise way whereby the PG and granule cells control the spiking of the mitral cell. Based on this finding we may deviate from the purely empirical approach adopted here and draw some conjectures on the possible roles of this stepwise control mechanism. The most direct one is that it may be beneficial to the stability of the system, making the response of the mitral cell more robust to variations in the maximum conductances of the synapses received from the inhibitory interneurons. On the other hand, if we assume the symmetric roles of the maximum conductance of the synapse made by the PG cell on the mitral cell (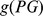) and the amplitude of the stimulus arriving at the PG cell (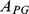) on the activity of the mitral cell suggested by the study of PG and mitral cells alone (Figure 3C) and extrapolate this symmetry to the case of the cell triad, we may associate to each frequency band of the mitral cell in the 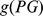 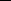 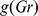 diagram a range of amplitudes of stimuli applied to the PG cell: high stimuli amplitudes would correspond to low frequencies and low stimuli amplitudes would correspond to high frequencies. One would have then a stepwise mechanism of coding the amplitude of a stimulus arriving on the PG cell (which is related to odorant identity) in which the step size would be determined by the amplitude of the stimulus and the maximum conductance of the inhibitory synapse of the granule cell. In such a mechanism, relevant for odorant coding, the granule cell could have a more important role than the one observed in the present study, especially in the context of a larger network in which lateral inhibitory effects would be expected to occur.

Another possible effect of the granule cell would be a fine control of the spike times of the mitral cell. Figure 5B shows that the firing frequency of the mitral cell is much more sentivive to small variations in 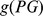 than in 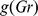. In this diagram there are aproximately horizontal frequency bands, determined by fixed 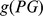 values, for which variations of 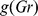 within certain ranges do not alter the firing frequency of the mitral cell. However, looking at Figure 5C one can see that, for the same fixed values of 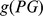, variations of 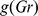 within the same ranges provoke small changes in the first spike time of the mitral cell. These spike time variations are not enough to change the firing frequency of the mitral cell but they may uniformize the phase cycle of the mitral cell. If this effect remains valid in a network context, the granule cells would have a role in controling the synchrony of the mitral cells [15].

Finally, it is important to note that our results are based on a model of an elementary local circuit found in the olfactory bulb and are, therefore limited to a study of the most basic effects of the inhibitory interneurons on the spiking of the mitral cell. A more complete investigation of the roles of these interneurons requires a network model. The PG cell and the triad model presented here will be used in the construction of a large-scale model of the olfactory bulb to further study the roles of the two interneuron types on olfactory processing.

## Materials and Methods

All simulations were run in NEURON version 7.2 [37] (http://www.neuron.yale.edu). The equations were integrated using the backward Euler method with a 0.05 ms time step. Parameter manipulations and data analysis were performed in Matlab (MathWorks, 1994–2010) by custom-made programs.

### Periglomerular Cell Model

**Figure 7:**
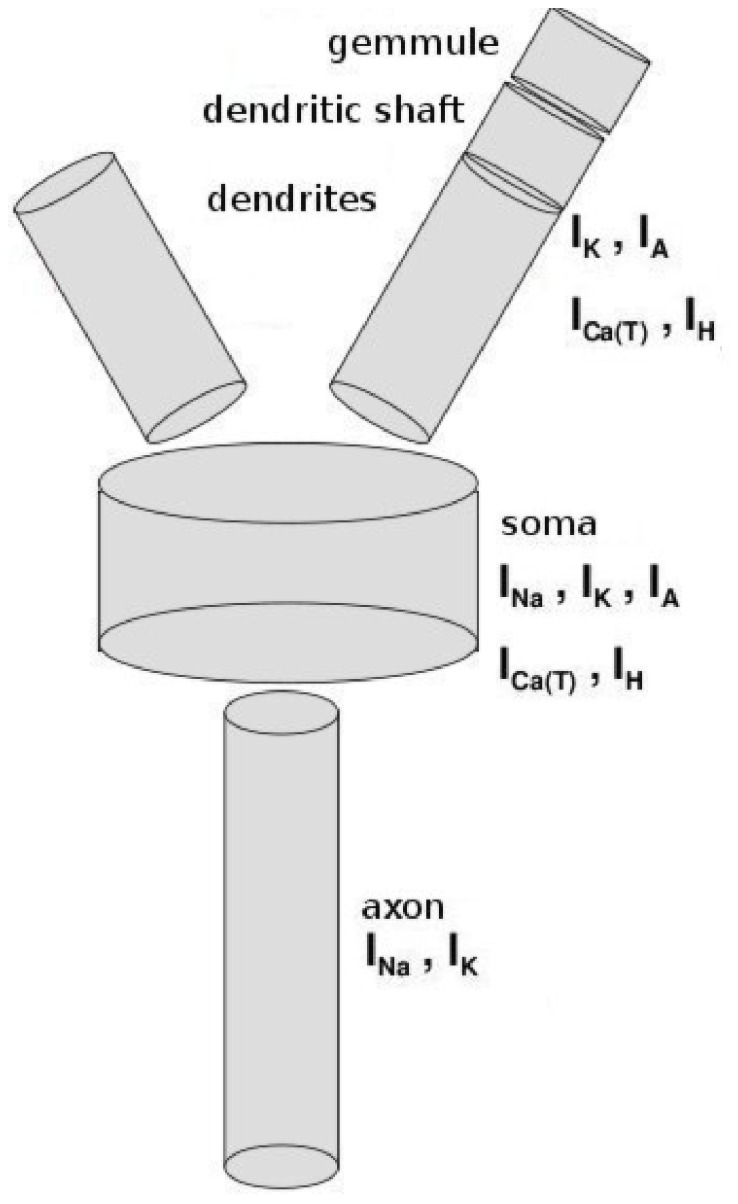
Scheme of the PG cell model. The names of compartments together with the membrane currents in them are placed beside the compartments. The currents are: sodium current (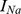), rectifier potassium current (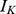), A-type inactivating potassium current (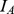), hyperpolarization-activated current (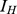), and low-thereshold T-type calcium current (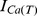).

The PG cell model is formed by six isopotential cylindrical compartments: soma (8 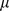
m length, 8 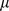
m diameter), axon (50 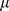
m length, 1 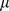
m diameter), two primary dendrites (20 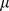
m length, 1 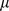
m diameter), dendritic shaft and gemmule (1 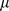
m length, 1 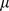
m diameter each). These compartments are connected as shown in Figure 7.

The PG cell model was constructed based on anatomical and biophysical data [21]–[23] and electrophysiological information about juxtaglomerular cells of the rat olfactory bulb [24]. The juxtaglomerular cells include different types of interneurons like PG cells, short axon cells and external tufted cells, and it is difficult to characterize each one of these cells experimentally. They are usually described collectively and so, whenever available, we used the generic parameters provided for these cells to construct our PG cell model.

The passive parameters of our model are: cytoplasmic resistivity (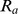) of 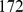 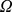 cm; total surface area of 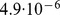 cm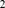; total capacitance (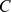) of 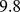 pF; and total membrane resistance (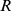) of 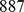 M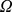. The resting membrane potential is 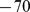 mV. These values were adjusted to replicate one of the few available electrophysiological recordings of the PG cell [24] and are compatible with experimental values reported elsewhere [27], [38]. It is known experimentally that juxtaglomerular cells have high input resistances, with values near 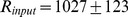 M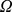 [24]. The input resistance we measured in our model was 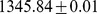 M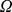.

The ionic currents incorporated into the model are also shown in Figure 7. The hyperpolarization-activated current (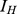) was detected in PG cells of the rat olfactory bulb by Cadette and Belluzzi [25]. In the same work they also gave the kinetic parameters for this current. The sodium (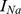), potassium (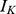) and calcium currents were investigated experimentally by Bardoni et al. [26], [27] and modeled by Destexhe et al. [39], [40]. The model of the low-thereshold T-type calcium current (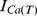) incorporates a calcium diffusion mechanism. The A-type inactivating potassium current (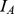) was detected by Bardoni et al. [27] and modeled by Migliore et al. [41]. These currents were added to our PG cell model keeping the kinetic parameters of the original models except the maximum conductance densities, which were adjusted in order to reproduce one of the experimental behaviors observed in cells of the juxtaglomerular layer of the olfactory bulb by McQuiston and Katz [24] (the one shown in Figure 1C of their paper). To simplify the adjustment of the maximum conductance densities we assumed each one to distributed uniformly over the compartments where they appear. The ionic currents were modeled according to the Hodgkin-Huxley formalism [42], and the models were constructed in the NMODL language [43]. The models for all ionic currents used in our PG cell model were made available in the ModelDB database [29] by their authors. A self-inhibition was incorporated in the model via a graded GABAergic self-synapse in the gemmule compartment.

To adjust the parameters of our PG cell model we submitted it to a depolarizing step current injection of 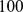 pA for 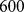 ms, reproducing the experimental protocol in [24]. The simulations were run for 1000 ms with stimulus onset occurring always at 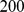 ms. The maximum conductance densities of the ionic currents and the conductance of the self-inhibitory synapse were adjusted to replicate the voltage curve given at Figure 1C of [24], which shows a single action potential of a juxtaglomerular cell in response to the stimulus protocol. The values of the parameters were varied over a parameter space encompassing about 50,000 combinations. The response generated by each combination was compared with the experimental voltage curve using the following error criterion [44]:
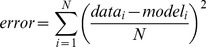
(1)where 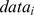 and 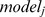 represent the membrane potential of the experiment and the model respectively; 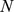 is the total number of data and model points.

In order to determine the best combination of parameters, we adopted the strategy of focusing on key features of the experimental response, such as spike time, action potential amplitude and membrane potential of the afterhyperpolarization plateau. So we used the following fitness function:
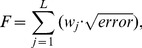
(2)where 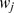 is the weight at different periods of time 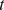 in a simulation of 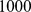 ms, given by
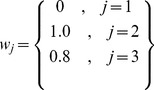
(3)where 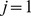 represents the no current injection period (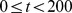 ms and 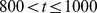 ms), 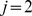 represents the duration of the spike (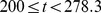 ms) and 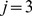 represents the period during which there is injected current (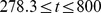 ms) after the end of the spike. So, the fitness function 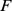 assigns higher weights to regions of interest of the response curve.

### Mitral and Granule Cell Models

The mitral and granule cell models used by us were taken from previous works by other authors, and their parameters and characteristics were kept as in the original models. The mitral cell model [14] is composed by four compartments, namely glomerular tuft, primary dendrite, soma and secondary dendrite. The granule cell model [15], [30] has three compartments, deep dendrite, soma and peripheral dendrite.

The passive parameters and the maximum conductance densities of the mitral and granule cell models are given in Tables 3 and 4 respectively.

**Table 3:** Mitral cell model parameters.

	Compartment				
Maximum conductance densities (S/cm 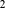 )	Soma	Glomerular tuft		Primary dendrite	Lateral dendrite
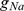	0.1532	0		0.00134	0.0226
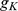	0.028	0.02		0.00174	0
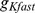	0.1956	0		0.00123	0.033
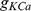	0.0142	0		0	0
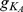	0.00587	0		0	0
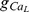	0.004	0.0095		0.0022	0
**Morfological parameters**					
Diameter ( 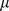 m)	16.2		26.7	104.4	170.9
Length ( 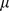 m)	100		100	100	100
Area ratio	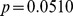		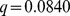	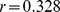	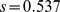

Parameters taken from Davison et al. [14].

**Table 4:** Granule cell model parameter.

	Compartment		
Maximum conductance densities (S/cm 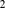 )	Soma	Peripheral dendrite	Deep dendrites
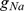	0.1611	0.1355	0
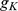	0.1313	0.0243	0
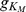	0.1334	0	0
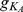	0.0088	0	0
**Morfological parameters**			
Diameter ( 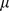 m)	0.72321	16.378	36.075
Length ( 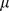 m)	50	50	50
Area ratio	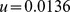	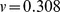	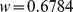

Parameters taken from Davison et al. [15].

### Synapses

The synapses incorporated into the model represent the synapses, usually reciprocal, found in the olfactory bulb [21]–[23]. The inhibitory synapses are mediated by GABA (gamma-aminobutyric acid) neurotransmitter and the excitatory synapses are mediated by AMPA (alpha-amino-3-hydroxy-5-methyl-4-isoxazol propionic) and NMDA (N-methyl-D-aspartate) glutamate receptors, which were combined into a single mechanism. The PG cell model has a self-inhibitory synapse made in the gemmule compartment, reproducing experimental evidence [8], [45].

All synaptic models were thresholded and graded to reflect the graded synaptic communication *in vivo*. We used the same model [28] for the excitatory and inhibitory synapses, with reversal potentials given by 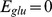 mV an 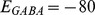 mV. All synaptic time constants were set to 1 ms.

### Cell Triad Model

**Figure 8:**
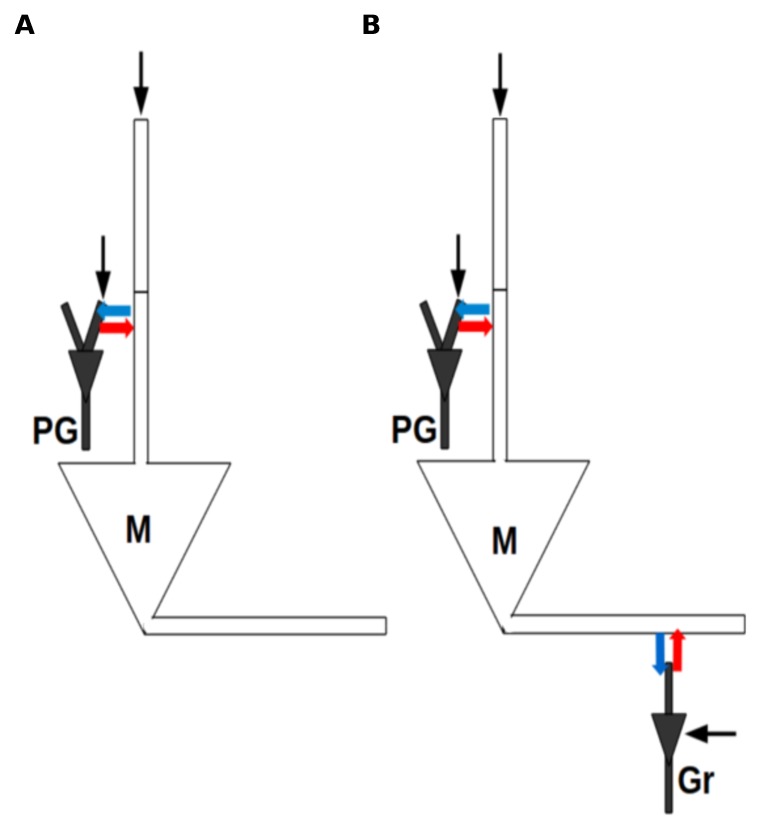
Scheme of connections in the cell triad model. The model is made of periglomerular (PG), mitral (M) and granular (Gr) cells. Blue arrows indicate excitatory synapses, red arrows indicate inhibitory synapses and black arrows indicate current inputs. (A) Simpler model with only PG and M cells. (B) Full cell triad model.

The reciprocal dendrodendritic synapses between the PG and mitral cells (Figure 8A) were implemented by coupling the primary dendrite compartment of the mitral cell and the gemmule compartment of the PG cell. On the other hand, the reciprocal dendrodendritic synapses between the mitral and granule cells (Figure 8B) were implemented by coupling the secondary dendrite compartment of the mitral cell and the peripheral dendrite compartment of the granule cell.

Besides these synaptic contacts, the cells of our model also receive external inputs in the form of injected current pulses. They represent inputs from olfactory receptor neurons in the case of the mitral and PG cells [14], [31], [46] and average stimuli from other olfactory bulb cells in the case of the granule cell [15]. Inputs to the mitral cell were aplied to its glomerular tuft compartment, inputs to the PG cell were applied to its gemmule compartment and inputs to the granule cell were applied to its soma compartment.
